# Climate Change Impact on Yield and Water Use of Rice–Wheat Rotation System in the Huang-Huai-Hai Plain, China

**DOI:** 10.3390/biology11091265

**Published:** 2022-08-25

**Authors:** Yanxi Zhao, Dengpan Xiao, Huizi Bai, De Li Liu, Jianzhao Tang, Yongqing Qi, Yanjun Shen

**Affiliations:** 1 Engineering Technology Research Center, Geographic Information Development and Application of Hebei, Institute of Geographical Science, Hebei Academy of Sciences, Shijiazhuang 050011, China; 2 College of Geography Science, Hebei Normal University, Shijiazhuang 050024, China; 3 Hebei Laboratory of Environmental Evolution and Ecological Construction, Shijiazhuang 050024, China; 4 NSW Department of Primary Industries, Wagga Wagga Agricultural Institute, Wagga Wagga, NSW 2650, Australia; 5 Climate Change Research Centre, University of New South Wales, Sydney, NSW 2052, Australia; 6 Key Laboratory for Agricultural Water Resources, Hebei Key Laboratory for Agricultural Water Saving, Center for Agricultural Resources Research, Institute of Genetics and Developmental Biology, Chinese Academy of Sciences, Shijiazhuang 050021, China; 7 School of Advanced Agricultural Sciences, University of the Chinese Academy of Sciences, Beijing 100049, China

**Keywords:** climate change, APSIM model, crop yield, agriculture water use, CMIP6

## Abstract

**Simple Summary:**

Quantitatively exploring the impact of climate change on crop production and water consumption (i.e., crop evapotranspiration during crop growth period, ET) is very important to achieve sustainable regional agricultural development. In this study, based on daily downscaled climate data from 22 Global Climate Models (GCMs), we applied the Agricultural Production Systems sIMulator (APSIM) to investigate the possible impact of climate change (e.g., temperature (*Temp*), solar radiation (*Rad*), precipitation (*Prec*) and CO_2_) on crop phenology, yield and water consumption for the rice (*Oryza sativa* L.) -wheat (*Triticum aestivum* L.) rotation. Due to the increase in *Temp*, the key phenological periods (flowering and maturity) of wheat in the future mainly tend to advance, while the phenological changes of rice show different trends at different sites. Both rice and wheat yields were negatively correlated with *Temp*, but positively correlated with *Rad*, *Prec,* and CO_2_ concentration ([CO2]). However, crop ET was positively correlated with *Rad*, but negatively correlated with [CO2], as elevated [CO2] decreased stomatal conductance. Moreover, the water use efficiency (WUE) of rice and wheat was negatively correlated with *Temp*, but positively correlated with [CO2]. Overall, climate change will have a significant impact on the crop growth process, yield and water consumption.

**Abstract:**

Global climate change has had a significant impact on crop production and agricultural water use. Investigating different future climate scenarios and their possible impacts on crop production and water consumption is critical for proposing effective responses to climate change. In this study, based on daily downscaled climate data from 22 Global Climate Models (GCMs) provided by Coupled Model Intercomparison Project Phase 6 (CMIP6), we applied the well-validated Agricultural Production Systems sIMulator (APSIM) to simulate crop phenology, yield, and water use of the rice–wheat rotation at four representative stations (including Hefei and Shouxian stations in Anhui province and Kunshan and Xuzhou stations in Jiangsu province) across the Huang-Huai-Hai Plain, China during the 2041–2070 period (2050s) under four Shared Socioeconomic Pathways (i.e., SSP126, SSP245, SSP370, and SSP585). The results showed a significant increase in annual mean temperature (*Temp*) and solar radiation (*Rad*), and annual total precipitation (*Prec*) at four investigated stations, except *Rad* under SSP370. Climate change mainly leads to a consistent advance in wheat phenology, but inconsistent trends in rice phenology across four stations. Moreover, the reproductive growth period (RGP) of wheat was prolonged while that of rice was shorted at three of four stations. Both rice and wheat yields were negatively correlated with *Temp*, but positively correlated with *Rad*, *Prec*, and CO_2_ concentration ([CO2]). However, crop ET was positively correlated with *Rad*, but negatively correlated with [CO2], as elevated [CO2] decreased stomatal conductance. Moreover, the water use efficiency (WUE) of rice and wheat was negatively correlated with *Temp*, but positively correlated with [CO2]. Overall, our study indicated that the change in *Temp*, *Rad*, *Prec,* and [CO2] have different impacts on different crops and at different stations. Therefore, in the impact assessment for climate change, it is necessary to explore and analyze different crops in different regions. Additionally, our study helps to improve understanding of the impacts of climate change on crop production and water consumption and provides data support for the sustainable development of agriculture.

## 1. Introduction

The global warming will become more severe throughout the 21st century as carbon dioxide (CO_2_) emissions continue to increase [1,2]. Currently, climate change and its impact are one of major research hotspots. Globally, climate change, characterized by climate warming, has had important impacts on agricultural production and consumption of agricultural water resources [3,4,5,6,7,8]. To some extent, the rise in temperature could accelerate the crop growth process and lead to shorter crop growth period, which are negatively influence the formation of crop yield and ultimately cause the crop failures [9,10]. Additionally, the interdependence of crop yield and crop water consumption is vital for sustainable agriculture [11,12,13]. The climate change would influence crop development period and thus the consumption of crop water resources undoubtedly [14]. Consequently, the assessment of the influence of climate change on agricultural production and consumption of agricultural water resources would be conducive to alleviating yield losses and agricultural sustainable development.

Recently, related studies have assessed the impact of climate change on agriculture over the past few decades [15,16]. With the deepening of research, scholars pay more attention to predicting the impact of climate change on crop production and agricultural water consumption [17,18,19,20]. The future climate scenarios of the most related studies are mainly from Global Climate Models (GCMs), which is a practicable tool to explore the mechanism related to climate change. The GCM projections of future climate generated can provide important data support for the studies about climate change at various scales [21]. Coupled Model Intercomparison Project Phase (CMIP) has been used for more than 20 years. The number of participating models for CMIP6 is the largest, the scientific experiment design is the most complete, and the largest number of models are provided as compared to climate models from previous CMIPs [22]. The physical mechanism (e.g., atmospheric radiation and deep convection process) of GCMs from CMIP6 is enhanced to make the GCMs more suitable for the projection of the climate distribution [23,24,25]. Therefore, the projection capacity of climate change for CMIP6 models has largely improved and the projections of CMIP6 models for climate systems were closer to the observed values and had less uncertainty than CMIP5 models [26,27,28].

The crop growth mechanism model (crop model) is a computer simulation program that can dynamically describe the process of crop growth, development, and yield formation under various environmental conditions by importing weather data, various parameters, soil data, and so on [29]. Many researchers have investigated the effects of climate change during the past few decades on crop phenology, yield, and evapotranspiration using various crop models to develop adaptive measures (such as adjustment of sowing date, renewal of crop variety, and optimization of cropping system) for reducing yield loss and improving water use [30,31,32]. Furthermore, the coupling of GCMs and crop models can be applied to the evaluation of the influence of future climatic variation on crop production and water consumption [33]. Generally, the change of climatic factors (e.g., solar radiation, temperature, and precipitation) in the region under future climate scenarios will change the crop growth process and yield formation mechanism [17]. Currently, the methods to improve water use efficiency (WUE) while maintaining high grain yield under future climate scenarios are very noteworthy, especially for the water-deficient region [13]. For example, the north of Huang-Huai-Hai Plain in China is facing the serious challenge of the crop water demand and groundwater overdraft during the wheat growth period. Additionally, long-term intensive irrigation has seriously affected the sustainable development of agricultural water resources in this area [34,35]. To some extent, adjusting cropping systems could be an effective measure to deal with the risks of climate change [36]. Nevertheless, most of the related studies are focused on the double cropping systems of winter wheat–summer maize, while there are fewer analyses about the effect of climate change on the rice–wheat rotation.

Wheat and rice are the most important staple cereal crop in China with a large planting area, wide distribution, and high grain yield [37]. The southern part of Huang-Huai-Hai plain is the traditional rice–wheat rotation area in China [38]. In this study, we selected future climate projections from 22 CMIP6 GCMs under four societal development pathways (SSP126, SSP245, SSP370, and SSP585) [39] to assess climate change and its impact on rice–wheat rotation system. Additionally, the Agricultural Production Systems Simulator model (APSIM), has been calibrated and validated based on observed experimental data for wheat and rice at four representative agro-meteorological stations. We used APSIM to simulate crop phenology, yield, and water use during the baseline period (1981–2010) and future period (2041–2070) under projected future climate scenarios. Our objectives were: (1) to simulate the impacts of climate change on crop yield and water use of rice–wheat rotation; and (2) to identify the contribution of climate changes to crop yield and water use. Our results should provide data support for the balance between crop production and crop water use to adopt adaptive measures under future climate scenarios.

## 2. Materials and Methods

### 2.1. Study Sites

**Figure 1:**
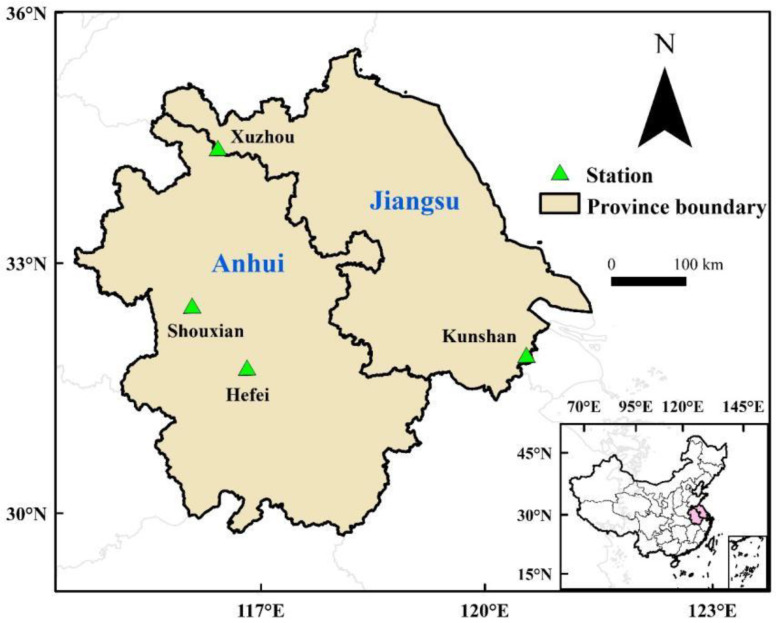
The spatial distribution of four selected agro-meteorological stations in the Huang-Huai-Hai Plain, China.

The study region includes Jiangsu and Anhui provinces, which is an important grain production region in China, where the main cropping system is the double-cropping systems of rice–wheat. In the study region, continuous broad irrigation was applied to rice, while wheat was rain-fed. Four agro-meteorological stations in the south of Huang-Huai-Hai Plain (HHHP) of China, including Hefei and Shouxian stations in Anhui province and Kunshan and Xuzhou stations in Jiangsu province, were selected for the study (Figure 1). Wheat is usually planted in middle or late October and harvested in late May or early June, while rice is grown from middle June to early October. This study region has a warm and humid monsoon climate with abundant light, heat resources, and precipitation. During 1981–2010, mean daily maximum and minimum temperature during wheat growth period ranged from 13.9 to 14.8 °C and from 4.3 to 6.7 °C, respectively; total precipitation and mean daily radiation ranged from 220 to 472.9 mm and from 10.2 to 11.1 MJ m^−2^, respectively (Figure S1). Mean daily maximum and minimum temperature during rice growth period ranged from 28.2 to 28.7 °C and from 19.4 to 21.1 °C, respectively; total precipitation and mean daily radiation ranged from 567.2 to 654.6 mm and from 14 to 14.9 MJ m^−2^, respectively (Figure S2).

### 2.2. Historical Climate Data and Future Climate Projections

We obtained the historical records about daily climate data, including mean temperature (*Tmean*), maximum temperature (*Tmax*), minimum temperature (*Tmin*), precipitation (*Prec*), and sunshine hours (*Sh*) from 1981 to 2010 for four agro-meteorological stations in China from China’s Meteorological Administration (CMA). Daily solar radiation (Rad) was calculated from the *Sh* using the Angstom–Prescott equation [40] as:

(1) $$\mathrm{Rad}=\left(a+b\frac{n}{N}\right)\times R_{a}$$

where R_a_ is extraterrestrial Rad (MJ m^−2^ d^−1^); n and N are actual and theoretical sunshine hours, respectively. The terms a and b are coefficients calibrated using observed radiation for the investigated stations [16].

Future climate scenario data were obtained from 22 GCMs (Table 1), which is provided by the World Climate Research Program (WCRP) of CMIP6 [41]. CMIP6 integrated climate change information from the CMIP5 simulations of the Representative Concentration Pathway (RCP) and future societal development pathways (SSPs), while the SSPs describe alternative evolutions of future society under climate change and/or climate policy [39]. Our study used future climate projections for four SSPs, including the combination of SSP1 and RCP2.6 (defined by SSP126), the combination of SSP2 and RCP4.5 (defined by SSP245), the combination of SSP5 and RCP8.5 (defined by SSP585) and a new scenario SSP3-7.0 (defined by SSP370). SSP126 is the updated RCP2.6 scenario, which represents combination of low mitigation stress, low social vulnerability, and low radiative forcing. SSP245 is the updated RCP4.5 scenario, which represents combination of medium social vulnerability and medium radiative forcing. SSP370 is a new scenario, which emphasizes the sensitivity of local climate change to land use and aerosol forcing and represents combination of high social vulnerability and relatively high radiative forcing. SSP585 is the updated RCP8.5 scenario, which represents combination of highest social vulnerability and highest radiative forcing. Further details of the SSPs can be found in the related studies [39,42]. All GCMs under four SSPs with a time span of 2040–2070 (2050s) were selected in this study.

We used the statistically downscaled method developed by Liu and Zuo [43] to generate daily climate data at each station from the monthly data of 22 GCMs. This method was mainly divided into two steps: spatial downscaling and temporal downscaling. The spatial downscaling was to transform monthly GCMs on the grid scale into monthly station data using the inverse distance-weighted interpolation (IDW). In the spatial downscaling process, we used qq-mapping bias correction method to correct the bias of the spatial downscaling data to match with the observations. Then, we transformed the spatial downscaling monthly climate data for each station into daily climate data using modified stochastic weather generator (WGEN) [44]. The future climate data outcomes generated by above method have been applied in many related studies [45,46].

### 2.3. Crop Data and APSIM Model

Detailed crop records included phenology (sowing date (SD), flowering date (FD), and maturity date (MD)), grain yield, and management data at the agro-meteorological experiment stations for 2005–2009 obtained from CMA. Additionally, reproductive growth period (RGP) was defined as the period from FD to MD. Water use efficiency (WUE) was defined as WUE = Yield/ET [47], where ET is crop evapotranspiration.

The APSIM model is a comprehensive model developed to simulate biophysical processes in agricultural systems [48,49]. Generally, APSIM model can provide acceptable prediction of crop productivity under the combined influences of climate change, soil condition, and management measures and is widely employed in agricultural research [50,51,52]. We selected the APSIM model to simulate crop phenology, yield, and water use during the baseline period (1981–2010) and future period (2041–2070) under future climate scenarios at the four selected stations. In our previous study, APSIM model was calibrated and validated based on observed phenology data and grain yield data from the rice–wheat rotation system at the selected stations [38,53].

**Figure 2:**
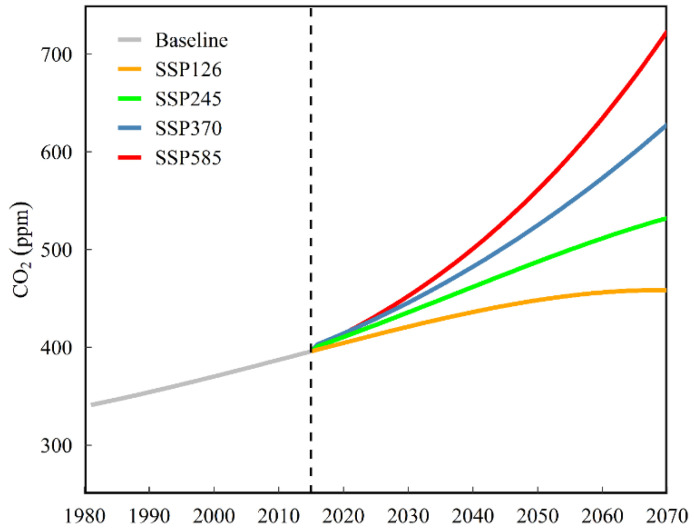
The atmosphere CO_2_ concentration under different future climate scenarios.

In APSIM model, radiation use efficiency, critical leaf nitrogen concentration, and transpiration efficiency are sensitive to the change in atmospheric CO_2_ concentration ([CO2]). With elevated atmosphere [CO2] under future scenarios, crop growth process can be affected significantly. We fitted the yearly atmosphere [CO2] under four SSPs in the future for integration into APSIM model using the empirical equation obtained by nonlinear least-squares regression [46]. Equations (2)–(5) were used to calculate the yearly atmosphere [CO2] for SSP126, SSP245, SSP370, and SSP585, respectively:

(2) $$\;{\left[\mathrm{CO}2\right]}_{y}=113.08-\frac{34.344-0.010402\times y}{0.15585-0.043727\times y^{0.28905}}+4.5948\times \left(y-1961\right)-0.023987\times {\left(y-1977\right)}^{2}-\frac{2.4959}{10^{4}}\times {\left(y-2054\right)}^{3}-\frac{6.5721}{10^{7}}\times {\left(y-2054\right)}^{4}\;\;\;\;\;\;\;\;$$

(3) $${\left[\mathrm{CO}2\right]}_{y}=62.044+\frac{34.002-3.8702\times y}{0.24423-1.1542\times y^{2.4901}}+0.028057\times {\left(y-1900\right)}^{2}+0.00026827\times {\left(y-1960\right)}^{3}-\frac{9.2751}{10^{7}}\times {\left(y-1910\right)}^{4}-2.2448\times \left(y-2030\right)\;\;$$

(4) $${\left[\mathrm{CO}2\right]}_{y}=151.9+\frac{20.092+10.315\times y}{5.8491+2.4884\times y^{2.268}}+\frac{4.752}{10^{5}}\times {\left(y-143.55\right)}^{2}+\frac{1.037}{10^{4}}\times {\left(y-1908\right)}^{3}-\frac{5.9113}{10^{8}}\times {\left(y-1849\right)}^{4}$$

(5) $${\left[\mathrm{CO}2\right]}_{y}=757.44+\frac{84.938-1.537\times y}{2.2011-3.8289\times y^{-0.45242}}+\frac{2.4712}{10^{4}}\times {\left(y+15\right)}^{2}+\frac{1.9299}{10^{5}}\times {\left(y-1937\right)}^{3}+\frac{5.1137}{10^{7}}\times {\left(y-1910\right)}^{4}\;\;$$

where *y* is the year for 1981–2070 (*y* = 1981, 1982, …, 2070). Figure 2 showed the atmosphere [CO2] under future scenarios calculated by above formulas.

## 3. Results

### 3.1. Projected Climate Change from GCMs

**Figure 3:**
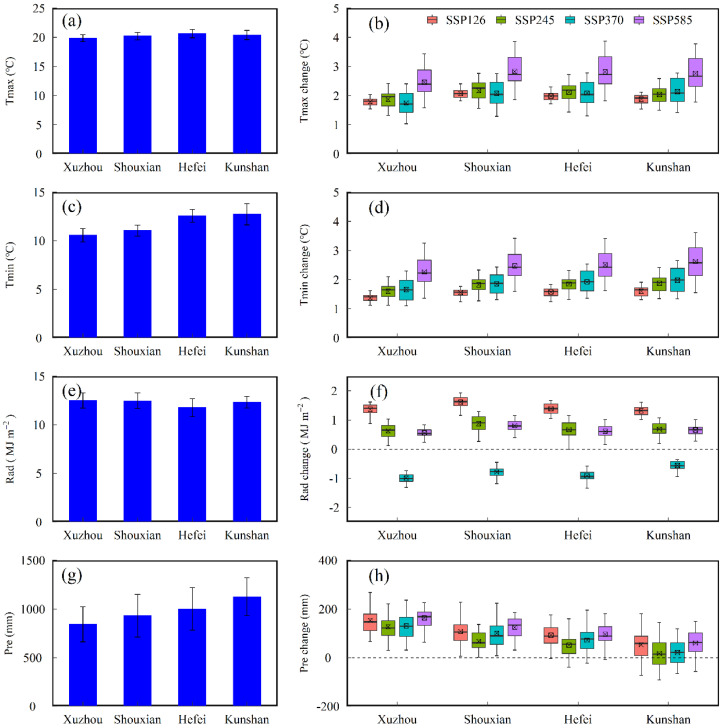
Annual mean maximum (*Tmax*) (**a**) and minimum temperature (*Tmin*) (**c**), solar radiation (*Rad*) (**e**) and precipitation (*Pre*) (**g**) for the baseline period (1981–2010) and projected changes in annual mean *Tmax* (**b**), *Tmin* (**d**), *Rad* (**f**) and *Pre* (**h**) in the 2050s (2041–2070) period under SSP126, SSP245, SSP370, and SSP585 compared to the baseline period across the 22 CMIP6 GCMs in the four agro-meteorological stations. The black lines and crosshairs within each box indicate the multi-model median and mean, respectively. Box boundaries indicate the 25th and 75th percentiles across 22 GCMs, whiskers below and above the box indicate the 10th and 90th percentiles. Same as below figures.

Annual mean *Tmax* and *Tmin*, *Rad,* and *Pre* for the baseline period (1981–2010) and projected changes in annual mean *Tmax* and *Tmin*, *Rad,* and *Pre* under future scenarios compared to the baseline period across the 22 GCMs in the four agro-meteorological stations were shown in Figure 3. The projected temperature (*Tmax* and *Tmin*) under future scenarios increased obviously across the four selected stations, especially under SSP585 scenario, with an average increase amplitude of over 2 °C (Figure 3b,d). For *Rad*, most projections from GCMs under SSP126, SSP245, and SSP585 increased significantly, while the mean upward magnitude under SSP126 scenario was more than 1 MJ m^−2^ and the change range under SSP245 and SSP585 scenarios were 0–1 MJ m^−2^ (Figure 3f). However, the projected values of *Rad* from GCMs under SSP370 scenario showed a decreasing trend compared to the baseline period (Figure 3f). There were increasing trends in the most of projected *Pre* changes under four future scenarios except for some GCM models at Hefei and Kunshan stations (Figure 3h). Additionally, the difference in variation ranges for *Pre* under different future scenarios was not marked, and there was no single scenario that was particularly prominent compared with other SSP scenarios (Figure 3h). The mean *Tmax* and *Tmin*, *Rad,* and *Pre* for the baseline period and projected changes in the mean *Tmax* and *Tmin*, *Rad,* and *Pre* under future scenarios compared to the baseline period across the 22 GCMs in the four agro-meteorological stations during rice and wheat growth period were shown in Figures S1 and S2, respectively.

### 3.2. Crop Phenology Change under Future Climate Scenarios

**Figure 4:**
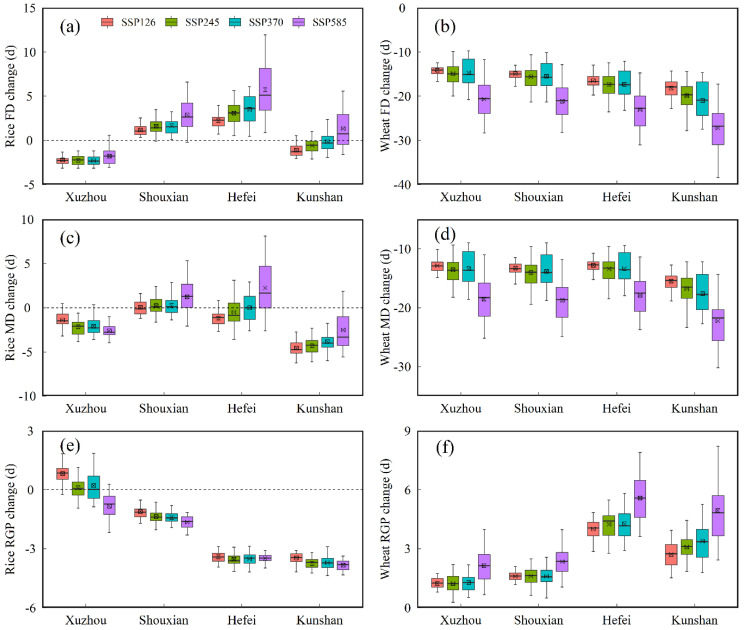
Projected changes in flower date (FD) (**a**,**b**), maturity date (MD) (**c**,**d**) and reproductive grow period (RGP) (**e**,**f**) for rice (**a**,**c**,**e**) and wheat (**b**,**d**,**f**) in the 2050s (2041–2070) period under SSP126, SSP245, SSP370 and SSP585 compared to the baseline period (1981–2010) across the 22 CMIP6 GCMs in the four agro-meteorological stations.

Future climate variation would accelerate the crop growth process and change the crop growth period. Figure 4 shows the projected changes in FD, MD, and RGP for rice and wheat under future scenarios compared to the baseline period across the 22 CMIP6 GCMs in the four agro-meteorological stations.

For rice, there was an advancing trend in FD and MD under future scenarios at the Xuzhou station, with the average variation range of 1.8–2.3 d and 1.4–2.6 d, respectively (Figure 4a,c). The FD and MD at Kunshan station also advanced in the 2050s except for the FD under SSP585 (Figure 4a,c). Comparatively speaking, the FD at Shouxian and Hefei stations showed a significantly delaying trend under future scenarios, while the change amplitude of MD in the future was not remarkable with the exception of the variation under SSP585 (Figure 4a,c). Moreover, the mean change magnitude of the RGP at Xuzhou station in the future was 0–1 d (Figure 4e). As compared with the Xuzhou station, the RGP at Shouxian, Hefei, and Kunshan stations shortened significantly, with the average decreasing interval of 1.1–1.7 d, 3.2–3.5 d, 3.5–3.9 d, respectively (Figure 4e).

For wheat, the FD and MD across all the stations under four SSP scenarios advanced obviously (Figure 4b,d). Meanwhile, the change amplitude in FD and MD under SSP585 scenario was more than in other scenarios (Figure 4b,d). The advancing ranges in FD across all the stations under future scenarios exceeded those in MD, which prolonged the RGP for wheat (Figure 4f). The average extended days in RGP under future scenarios at Xuzhou and Shouxian stations ranged from 1.2 to 2.3 d, while the RGP under future scenarios at Hefei and Kunshan stations increased by 2.7–5.6 d (Figure 4f). Therefore, the increasing trend in RGP under SSP585 scenario was particularly significant in comparison with other SSPs (Figure 4f).

### 3.3. Grain Yield Change under Future Climate Scenarios

**Figure 5:**
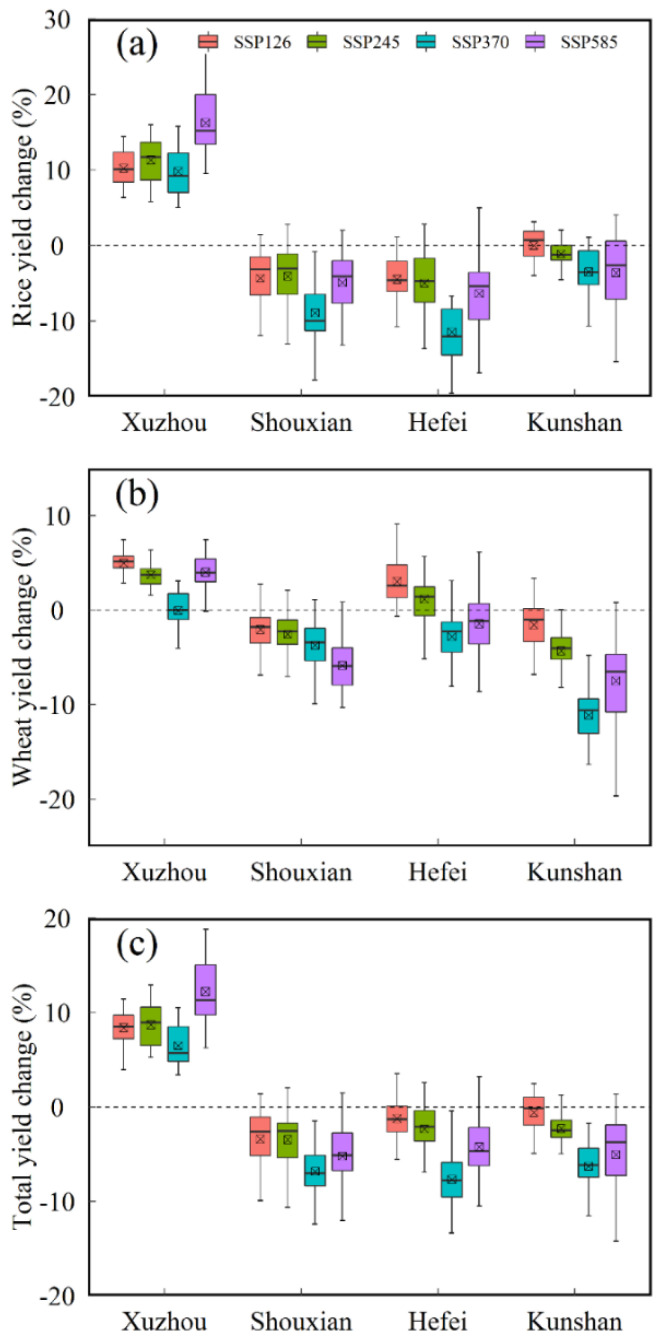
Projected changes in yield for rice (**a**), wheat (**b**), and total of rice and wheat (**c**) in the 2050s (2041–2070) period under SSP126, SSP245, SSP370, and SSP585 compared to the baseline period (1981–2010) across the 22 CMIP6 GCMs in the four agro-meteorological stations.

Climate change will change the crop growth period and have a significant impact on crop yield formation. The projected changes in yield for rice, wheat, and a total of rice and wheat under future scenarios compared to the baseline period across the four agro-meteorological stations were depicted in Figure 5. Additionally, we used regression analysis to reflect the relationship between crop (rice, wheat, and total of rice and wheat) yield change and climatic variables variation such as *Tmean*, *Rad*, *Pre,* and [CO2] (Table 2). 

The yield change for rice under future scenarios at Xuzhou station increased by 9.8–16.3%, while the decline magnitude of rice yield in the 2050s at Shouxian, Hefei, and Kunshan stations was 4.1–8.9%, 4.5–11.4%, and 0–3.6%, respectively (Figure 5a). This can be due to the difference in RGP for rice at different stations (Figure 4e). For wheat, there was an upward trend in yield under future scenarios at Xuzhou station, with an increasing interval of 0–5% (Figure 5b). However, wheat yield in the future at other stations decreased aside from yield at Hefei station under SSP126 and SSP245, though the RGP for wheat at three stations increased (Figure 5b). We speculate that the negative effects of temperature change on crops may be the main reason (Table 2). The total yield of rice and wheat under future scenarios at Xuzhou station increased by 6.5–12.3%, while the total yield of rice and wheat at other stations declined by 0.6–7.7% (Figure 5c).

As shown in Table 2, there was a significant correlation between crop (rice, wheat, and total of rice and wheat) yield change and climatic variables change other than the relation between rice yield and the *Pre* during the rice growth period. In general, crop (rice, wheat, and total of rice and wheat) yield was negatively correlated with *Tmean*, but positively correlated with *Rad*, *Pre,* and [CO2].

### 3.4. Crop Water Use under Future Climate Scenarios

**Figure 6:**
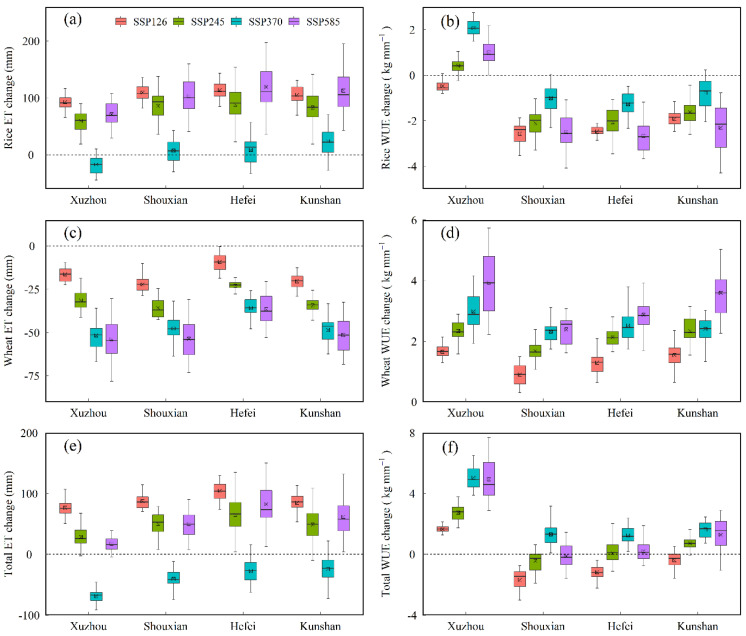
Projected changes in ET (**a**,**c**,**e**) and WUE (**b**,**d**,**f**) for rice (**a**,**b**), wheat (**c**,**d**) and total of rice and wheat (**e**,**f**) in the 2050s (2041–2070) period under SSP126, SSP245, SSP370 and SSP585 compared to the baseline period (1981–2010) across the 22 CMIP6 GCMs in the four agro-meteorological stations.

There was a considerable impact of climate change on the photosynthesis and yield formation mechanisms in crops. We used 22 CMIP6 GCMs in the 2050s under SSP126, SSP245, SSP370, and SSP585 scenarios to project the changes in ET and WUE for rice, wheat, and a total of rice and wheat compared to the baseline period (1981–2010) at the four selected stations (Figure 6). The rice ET in the future across the stations increased obviously except for that under the SSP370 scenario (Figure 6a). The rice WUE under future scenarios at Shouxian, Hefei, and Kunshan stations decreased significantly, but the changing trend of WUE at Xuzhou was the opposite (Figure 6b), which was mainly because of the difference in rice yield change (Figure 5a). The average decline magnitude for wheat ET across the stations increased in a sequence of SSP126, SSP245, SSP370, and SSP585 scenarios, which was 9.1–22.2 mm, 23–35.8 mm, 35.8–51.6 mm, and 36.6–53.8 mm, respectively (Figure 6c). Furthermore, the mean increasing amplitude for wheat WUE across the stations increased in order of SSP126, SSP245, SSP370 and SSP585 scenarios, which was 0.9–1.7 kg mm^−1^, 1.7–2.4 kg mm^−1^, 2.3–3.0 kg mm^−1^ and 2.4–4.0 kg mm^−1^, respectively (Figure 6d). For total of rice and wheat, the ET in the future at all the stations showed a significantly upward trend other than that under the SSP370 scenario (Figure 6e). The WUE under future scenarios at Xuzhou station increased remarkably, with the change interval of 1.7–5.1 kg mm^−1^, while the variation magnitude in WUE at other stations was relatively limited (Figure 6f).

In the meantime, we analyzed the impact of climatic variable change on the ET and WUE (Table 2). The ET for rice and total ET of two crops were positively correlated with *Tmean* and *Rad* but negatively correlated with *Pre* and [CO2]. Conversely, wheat ET was positively correlated with *Rad* but negatively correlated with [CO2]. Further, the WUE for wheat, rice and total WUE of two crops were negatively correlated with *Tmean* but positively correlated with [CO2].

## 4. Discussion

### 4.1. Impact of Climate Change on Crop Phenology

Generally, climate warming can accelerate crop growth process and has a significant impact on crop phenology, yield accumulation, and water consumption [14,54]. In our study, the rise in temperature advanced the FD and MD for wheat, while the difference of advance amplitude led to a longer RGP for wheat. These results are consistent with the conclusions of some related studies [46,55,56]. The variation of FD and MD for rice ranged from 0 to 5 d, while the change magnitude of FD and MD for wheat was more than 10 d. We suspect that the warm winter that shortened the overwintering period of wheat may be the main reason [57]. Climate warming can change crop growth and shorten the growth stages of rice, such as the vegetative growth period (VGP) and reproductive growth period (RGP) [58]. The RGP for rice simulated by crop model under future scenarios in our study showed a significantly decreasing tendency at most of the stations except for the Xuzhou station.

### 4.2. Impact of Climate Change on Crop Yield

The change in crop growth period can exert a significant influence on crop yield formation [36,54,56]. In our study, the response of crop growth to climate and environmental conditions at distinct stations was inconsistent. Xuzhou station, as a northern station, was different in the projected change of crop phenology and yield from other southern stations (Shouxian, Hefei, and Kunshan stations). The variation trend of RGP for rice and wheat at Xuzhou station was consistent with that of yield for rice and wheat with the exception of rice under SSP585. At other southern stations, the decline of the RGP for rice during the future period was in accordance with the change in rice yield. Nevertheless, the extension of the RGP for wheat under future scenarios was contrary to the decrease in wheat yield. We suspect that the negative effect of elevated temperature on yield may be the main factor. The temperature during the flowering and grain-filling period for wheat during 1981–2009 at southern stations was close to or above the optimum temperature [57]. The warm temperatures can improve the growth of crops before the temperature reaches the threshold, but yields will abruptly diminish subsequently [59,60]. Moreover, the sensitivity to high-temperature changes at different growth stages of the crop [61], was particularly significant during the RGP [62]. The high temperature during the RGP can affect pollination, reduce male fertility and seed quality, and ultimately lead to the loss of kernel weight and yield [62,63,64,65].

Solar radiation is the energy source of crop growth, which is vital for crop yield formation [46,66,67]. In our study, solar radiation was positively correlated with crop (rice and wheat) yield. Additionally, some studies obtained similar conclusions [47,56,68]. It was worth noting that the projected change (increased or decreased) in yield under the SSP370 scenario was less than in other SSP scenarios. This is due largely to the reduction in radiation under the SSP370 scenario. SSP370 is a new radiative forcing scenario, with high aerosol emissions and high air pollutant emissions, such as CH_4_, SO_2,_ and black carbon (BC) [69,70]. The high emissions of aerosol and air pollutants would lead to the reduction of radiation and the increase in foggy days [71]. The decline in solar radiation would attenuate photosynthesis, reduce dry matter accumulation time and cause yield loss for crops. The change in precipitation has little effect on rice yield [72], but has a significant effect on wheat yield [73]. We found that precipitation was positively correlated with the wheat crop, while the positive correlation between rice yield and precipitation was not significant. Additionally, the rise of [CO2] has a better fertility effect on crop growth, which can promote photosynthesis and increase crop yield [74,75,76,77,78]. Our regression results showed that crop yield was positively correlated with [CO2].

### 4.3. Impact of Climate Change on Crop Water Use

In general, the rise in temperature can shorten the crop growth period and reduce water consumption of crops [47]. However, we found that temperature was positively related to crop ET. We suspect that this is probably mainly because the elevated temperature can benefit the photosynthetic mechanism of crop and increase crop water consumption [46]. Additionally, solar radiation can improve the photosynthetic efficiency similar to temperature [79], while the rising [CO2] could reduce stomatal conductance and restrain the increase in water consumption [74]. We found that solar radiation was positively correlated to crop ET, while [CO2] was the opposite. Our regression analysis indicated that the change in precipitation has little effect on crop ET, which may be due to sufficient precipitation and less crop water stress in the study area. Under the comprehensive influence of future climate factors (including temperature, solar radiation, precipitation) and [CO2], rice ET would increase, and wheat ET would decrease. Eventually, the WUE of wheat increased and the WUE of rice declined except for Xuzhou station. The main reason may be that the yield of rice at Xuzhou station in the future increased significantly. 

Overall, as the increase in ET in rice was greater than the decline in ET in wheat, the total ET of rice and wheat showed an upward trend under future scenarios aside from the SSP370 scenario. The decline in solar radiation under the SSP370 scenario can weaken photosynthesis, inhibit the growth process of crops, reduce the water consumption of crops, and eventually lead to a significant decrease in the dry matter accumulation of crops above ground [47,56]. Many studies reported that the crop growth process and productivity in different regions vary greatly due to the differences in climate conditions and management levels in different regions [57,80,81]. We found that the increase in total yield at the northern station under future scenarios was larger than that at other southern stations, which led to the higher WUE at the northern station. Furthermore, water resources are abundant in the south of the HHHP, and the contradiction between high water consumption and crop grain yield in the south of the HHHP is not as acute as that in the north of the HHHP. Therefore, our future research direction will be on how to reduce the adverse effects of heat stress, heavy precipitation, drought, and weak radiation on crop productivity in this region.

### 4.4. Uncertainty and Limitation of the Study

There is still some uncertainty and limitation in our study. Firstly, the adoption of adaptation strategies to cope with climate change is not considered. Adaptation strategies, including the adjustment of sowing date and fertilizer rate, cultivar shift, and so on, are of great significance to crop growth under climate change scenarios, which can effectively offset the adverse effect of climate change on crop growth [47,82,83]. Secondly, we do not consider the impact of extreme weather conditions on crop production. The effect of extreme climate events (drought, heavy precipitation, heat damage, frost, etc.), on crop production, is far greater than that of the average climate change [59,73,84,85], while there are remarkable limitations for the crop model in response to the extreme climate events [86,87]. It would be an important research direction to consider comprehensively the effects of average climate and extreme climate on crop productivity based on crop models in the future [45]. Finally, the results of the crop model are uncertain under diverse environmental conditions due to the complex model structure and numerous input parameters in the crop model. Alternatively, the model performance can be enhanced by improving the model’s structure [88]; on the other hand, the uncertainty of the crop model can be reduced by multi-model ensemble [89]. We aim to improve the deficiencies in the next study based on the above summary.

## 5. Conclusions

The research into the influence of climate change on crop production and water use could be useful for agricultural sustainable development. We predicted the projected change in crop phenology, yield, and water consumption for the rice–wheat rotation under future scenarios by using future climate data from 22 CMIP6 GCMs. Overall, the FD, MD, and RGP for rice and wheat changed greatly in the future due to the effects of climatic variation. Additionally, climate change would have a significant impact on crop production. Crop yield was negatively correlated with temperature but positively correlated with solar radiation, precipitation, and [CO2]. In addition, climate change had a considerable influence on crop water consumption. Under the comprehensive influence of future climate factors, we predict that the ET and WUE of crops would change remarkably. There was a significant positive correlation between solar radiation and ET, while the ET of crops was negatively correlated with [CO2]. Moreover, the WUE of crops was negatively correlated with temperature but positively correlated with [CO2]. It is beneficial to develop adaptation strategies to alleviate or mitigate the negative effects of climate change on crop productivity and water resource use.

## Figures and Tables

**Table 1:** List of the 22 GCMs for future climate projections used in the study.

Code	GCM Name	Abbreviation	Institute ID	Country
1	ACCESS-CM2	ACC1	CSIRO–ARCCSS	Australia
2	ACCESS-ESM1-5	ACC2	CSIRO–ARCCSS	Australia
3	BCC-CSM2-MR	BCC	BCC	China
4	CanESM5	CAN1	CCCMA	Canada
5	CanESM5-CanOE	CAN2	CCCMA	Canada
6	CNRM-ESM2-1	CNR1	CNRM	France
7	CNRM-CM6-1	CNR2	CNRM	France
8	CNRM-CM6-1-HR	CNR3	CNRM	France
9	EC-Earth3	ECE1	EC–EARTH	Europe
10	EC-Earth3-Veg	ECE2	EC–EARTH	Europe
11	FGOALS-g3	FGO	FGOALS	China
12	GFDL-ESM4	GFD2	NOAA–GFDL	America
13	GISS-E2-1-G	GIS	NASA–GISS	America
14	INM-CM4-8	INM1	INM	Russia
15	INM-CM5-0	INM2	INM	Russia
16	IPSL-CM6A-LR	IPS	IPSL	France
17	MIROC6	MIR1	MIROC	Japan
18	MIROC-ES2L	MIR2	MIROC	Japan
19	MPI-ESM1-2-HR	MPI1	MPI-M	Germany
20	MPI-ESM1-2-LR	MPI2	MPI-M	Germany
21	MRI-ESM2-0	MRI	MRI	Japan
22	UKESM1-0-LL	UKE	MOHC	UK

**Table 2:** Coefficients in the regression analysis for the impacts of climate change on yield, ET, and WUE change.

Crop	a	b	c	d	R^2^
Δ*Yield* = aΔ*Tmean* + bΔ*Rad* + cΔ*Pre* + dΔ*[CO2]*					
Rice	−2455.17 **	562.05 **	0.44	20.35 **	0.46
Wheat	−392.47 **	173.95 **	2.73 **	1.26 **	0.36
Total	−3327.72 **	958.43 **	3.26 **	23.19 **	0.49
Δ*ET* = aΔ*Tmean* + bΔ*Rad* + cΔ*Pre* + dΔ*[CO2]*					
Rice	50.38 **	36.41 **	−0.02 *	−0.13 **	0.96
Wheat	2.20	4.01 **	0.01	−0.19 **	0.77
Total	48.32 **	50.16 **	−0.09 **	−0.23 **	0.89
Δ*WUE* = aΔ*Tmean* + bΔ*Rad* + cΔ*Pre* + dΔ*[CO2]*					
Rice	−4.49 **	0.11	0.01	0.03 **	0.68
Wheat	−0.8 **	0.14 *	0.01	0.02 **	0.66
Total	−6.19 **	0.48 **	0.01 **	0.05 **	0.59

Note: Shown in the table are the yield change (Δ*Yield*, kg ha^−1^), ET change (Δ*ET*, mm), and WUE change (Δ*WUE*, kg mm^−1^) as a function of mean temperature (Δ*Tmean*, °C), solar radiation (Δ*Rad*, MJ m^−2^), precipitation (Δ*Pre*, mm) and CO_2_ concentration change (Δ*[CO2]*, ppm). * and ** indicate significant at *p* < 0.05 and *p* < 0.01, respectively.

## Data Availability

The climatological data that support the findings of this study during manuscript preparation are available from the corresponding author on reasonable request. Detailed access to the data: The historical records about daily climate data from China’s Meteorological Administration (CMA) (http://data.cma.cn/, accessed on 12 February 2022); Future climate scenario data were provided by the World Climate Research Program (WCRP) of Coupled Model Inter-comparison Project phase 6 (CMIP6, https://esgf-node.llnl.gov/search/cmip6/ (accessed on 21 February 2022)).

## Supplementary Materials

The following supporting information can be downloaded at: www.mdpi.com/article/10.3390/biology11091265/s1, Figure S1: The mean maximum (*Tmax*) (a) and minimum temperature (*Tmin*) (c), solar radiation (*Rad*) (e) and precipitation (*Pre*) (g) for the baseline period (1981–2010) and projected changes in the mean *Tmax* (b), *Tmin* (d), *Rad* (f) and *Pre* (h) in the 2050s (2041–2070) period under SSP126, SSP245, SSP370, and SSP585 compared to the baseline period across the 22 CMIP6 GCMs in the four agro-meteorological stations during rice growth period; Figure S2: The mean maximum (*Tmax*) (a) and minimum temperature (*Tmin*) (c), solar radiation (*Rad*) (e) and precipitation (*Pre*) (g) for the baseline period (1981–2010) and projected changes in the mean *Tmax* (b), *Tmin* (d), *Rad* (f) and *Pre* (h) in the 2050s (2041–2070) period under SSP126, SSP245, SSP370 and SSP585 compared to the baseline period across the 22 CMIP6 GCMs in the four agro-meteorological stations during wheat growth period.
