# Supplementary material for: Climate Change Impact on Yield and Water Use of Rice–Wheat Rotation System in the Huang-Huai-Hai Plain, China
Source: Biology (Basel). 2022 Aug 25;11(9):1265. doi: 10.3390/biology11091265 (PMC9495956; doi:10.3390/biology11091265)
Supplement: Supplementary file 1 [file biology-11-01265-s001.zip › biology-1823039-supplementary.pdf]

**Supplementary Materials**

# Future climate change impact on yield and water use of rice – wheat rotation system in the Huang-Huai-Hai Plain, China

Yanxi Zhao[1,2,3], Dengpan Xiao[1,2,3*], Huizi Bai[3*], De Li Liu[4,5] and Jianzhao Tang[3]

[1] College of Geography Science, Hebei Normal University, Shijiazhuang 050024, China

[2] Hebei Laboratory of Environmental Evolution and Ecological Construction, Shijiazhuang 050024, China

[3] Engineering Technology Research Center, Geographic Information Development and Application of Hebei, Institute of Geographical Science, Hebei Academy of Sciences, Shijiazhuang 050011, China

[4] NSW Department of Primary Industries, Wagga Wagga Agricultural Institute, Wagga Wagga, New South Wales 2650, Australia

[5] Climate Change Research Centre, University of New South Wales, Sydney, New South Wales 2052, Australia

**Contents:**

**Figure S1. The mean maximum (*Tmax*) (a) and minimum temperature (*Tmin*) (c), solar radiation (*Rad*) (e) and precipitation (*Pre*) (g) for the baseline period (1981–2010) and projected changes in the mean *Tmax* (b), *Tmin* (d), *Rad* (f) and *Pre* (h) in the 2050s (2041–2070) period under SSP126, SSP245, SSP370 and SSP585 compared to the baseline period across the 22 CMIP6 GCMs in the four agro-meteorological stations during rice growth period.**

**Figure S2. The mean maximum (*Tmax*) (a) and minimum temperature (*Tmin*) (c), solar radiation (*Rad*) (e) and precipitation (*Pre*) (g) for the baseline period (1981–2010) and projected changes in the mean *Tmax* (b), *Tmin* (d), *Rad* (f) and *Pre* (h) in the 2050s (2041–2070) period under SSP126, SSP245, SSP370 and SSP585 compared to the baseline period across the 22 CMIP6 GCMs in the four agro-meteorological stations during wheat growth period.**

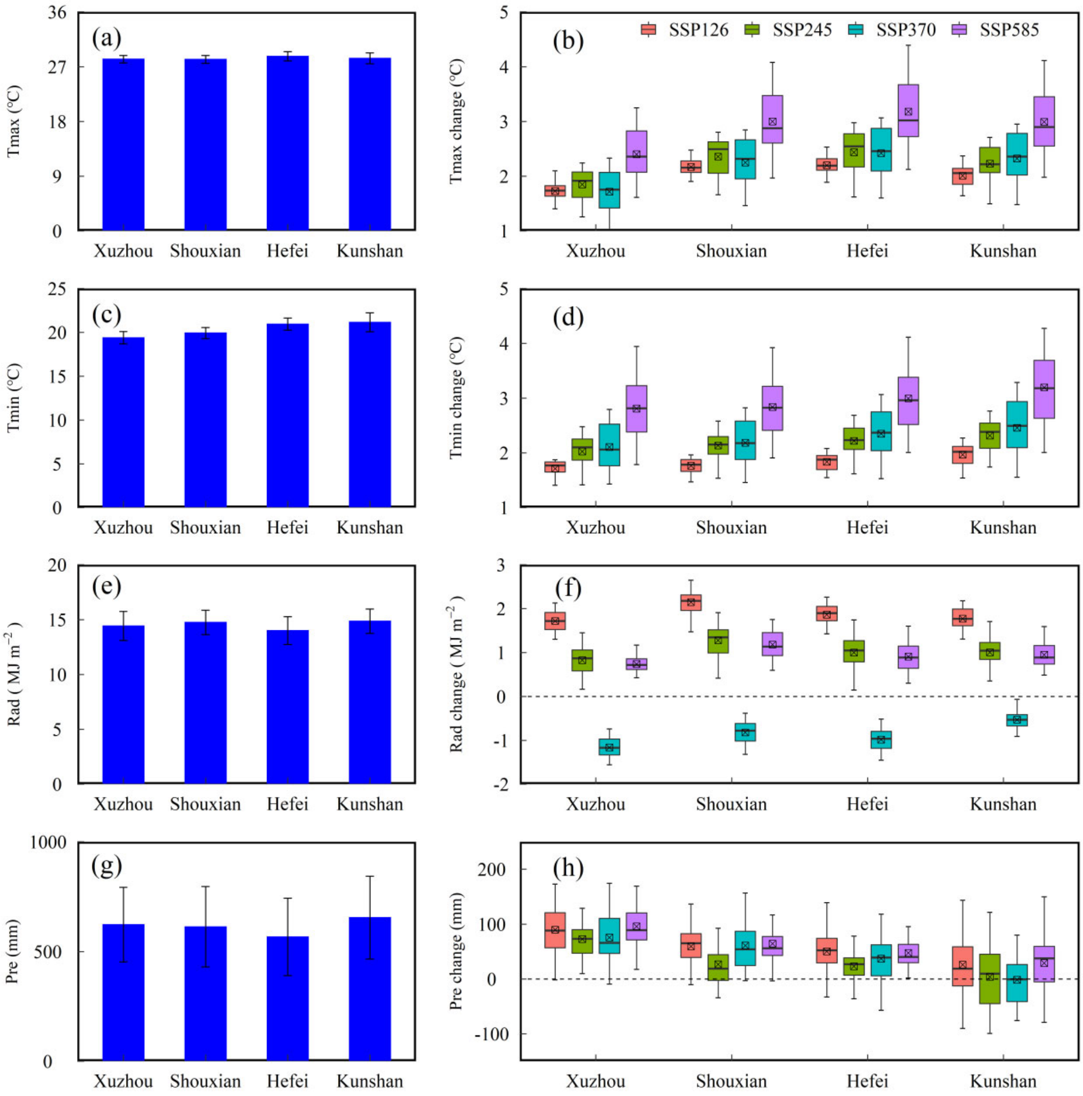


**Figure S1.** The mean maximum (*Tmax*) (a) and minimum temperature (*Tmin*) (c), solar radiation (*Rad*) (e) and precipitation (*Pre*) (g) for the baseline period (1981–2010) and projected changes in the mean *Tmax* (b), *Tmin* (d), *Rad* (f) and *Pre* (h) in the 2050s (2041–2070) period under SSP126, SSP245, SSP370 and SSP585 compared to the baseline period across the 22 CMIP6 GCMs in the four agro-meteorological stations during rice growth period.

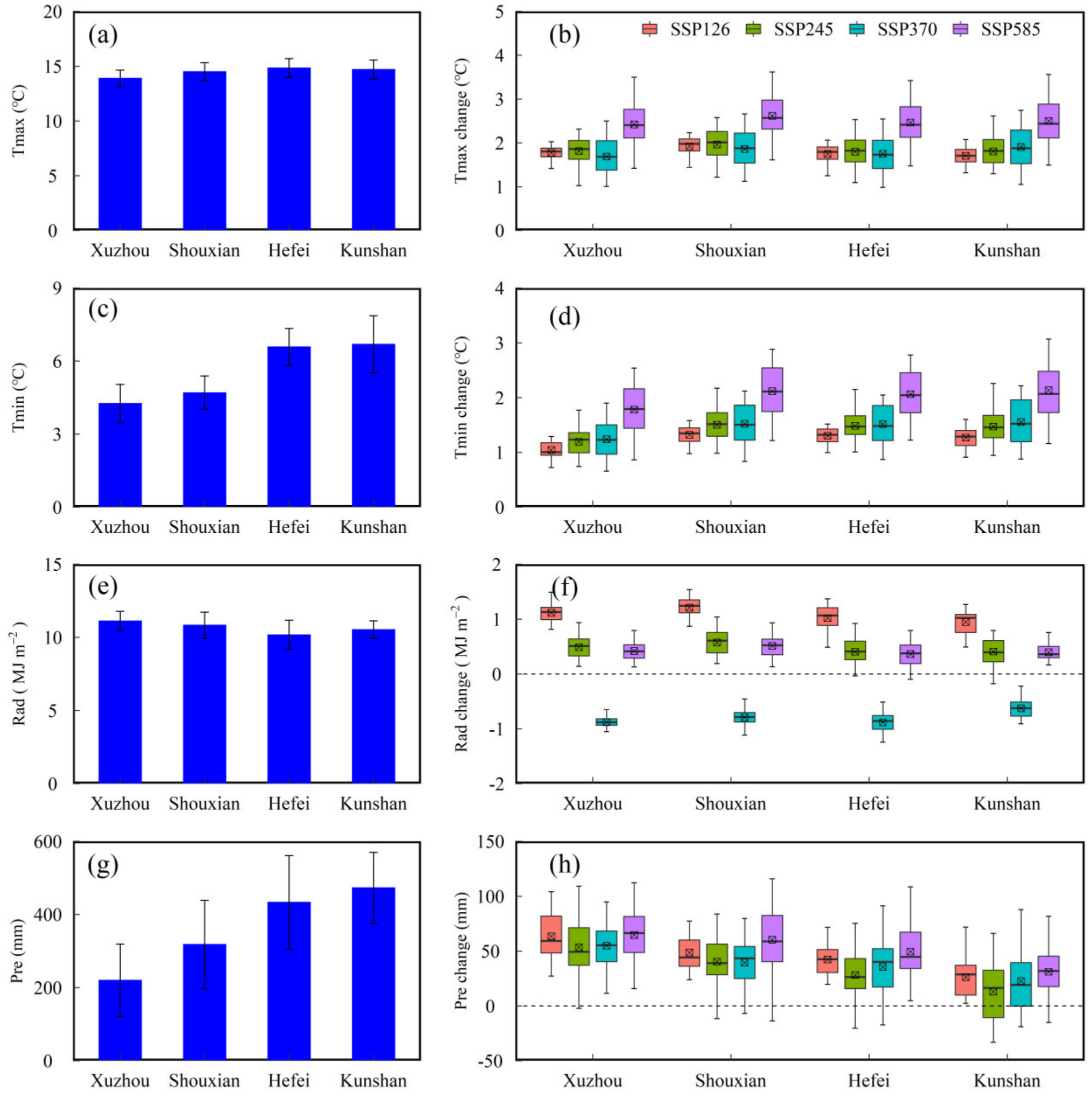


**Figure S2.** The mean maximum (*Tmax*) (a) and minimum temperature (*Tmin*) (c), solar radiation (*Rad*) (e) and precipitation (*Pre*) (g) for the baseline period (1981–2010) and projected changes in the mean *Tmax* (b), *Tmin* (d), *Rad* (f) and *Pre* (h) in the 2050s (2041–2070) period under SSP126, SSP245, SSP370 and SSP585 compared to the baseline period across the 22 CMIP6 GCMs in the four agro-meteorological stations during wheat growth period.
